# Soil fungal guilds as important drivers of the plant richness–productivity relationship

**DOI:** 10.1111/nph.16523

**Published:** 2020-03-28

**Authors:** S. Emilia Hannula, Sabrina Träger

**Affiliations:** ^1^ Department of Terrestrial Ecology Netherlands Institute of Ecology (NIOO‐KNAW) Droevendaalsesteeg 10 Wageningen 6708 PB the Netherlands; ^2^ Department of Botany Institute of Ecology and Earth Sciences University of Tartu Lai 40 Tartu 51005 Estonia

**Keywords:** fungal groups, herbaceous plant communities, mycorrhizal fungi, plant pathogenic fungi, saprotrophic fungi, soil fungal communities, soil–plant feedback

## Abstract

This article is a Commentary on https://doi.org/10.1111/nph.16390.

Researchers have long stewed over the relationship between plant species richness and productivity (i.e. aboveground biomass production) and its regulating processes in plant communities. Until now, mixed empirical evidence in experimental and natural studies still leaves many questions about the generality of any single relationship pattern and the underlying mechanisms. Plants are sessile and an enormous portion of their life is located belowground, in particular for herbaceous communities (Mokany *et al.*, 2006). Consequently, a closer look at the belowground environment of plants is due, but the majority of studies to date still focus on the aboveground environment. In this issue of *New Phytologist*, Chen *et al.* (2020; pp. 1129–1143) tackled the challenge to shed more light on the complex interaction network of plant species richness and productivity, considering the soil fungal community in contrasting soil fertility (i.e. soil nutrient level) environments. Chen *et al*. show, using two native transects of plant richness with contrasting soil fertility levels, that in soils with different nutrient levels, different fungal guilds interact with plants and with each other, which consequently leads to differences in the plant richness–productivity relationship. The novelty of this study lies within the focus on the whole rather than only one part of the spectrum of functional fungal groups – saprotrophic, mycorrhizal, and pathogenic – and their interrelated effect on plant richness and productivity in natural grasslands.

‘Assigning fungi into functional guilds [...] is definitely the way forward in understanding how soil‐inhabiting communities affect plant community dynamics and functions.’

Traditionally, plant species richness has been investigated as a response to productivity in herbaceous plant communities. The original and still most widely accepted pattern is that of a hump‐shaped model with plant species richness exhibiting an optimum at medium productivity and being limited at low and high productivity by high environmental stress and competition, respectively (Grime, 1973; Fraser *et al.*, 2015). Ever since, studies have challenged this approach by reporting the full range of relationship patterns (nonsignificant, linear, and unimodal) at the local, regional and global scale (Huston, 1979; Adler *et al.*, 2011). The reason for such variation can be manifold, ranging from differences in methodology, scale, site‐specific land‐use history, or simply due to site‐specific abiotic and biotic effects. It appears likely that neither one – plant richness nor productivity – influences the other by a simple direct path, but that their relationship is rather the result of a complex network of interactions on each variable with the aboveground and belowground abiotic and biotic environment. Therefore, another approach to qualify the richness–productivity relationship might be by disentangling indirect paths that act on either part in addition to the combination of richness and productivity. Among important factors are abiotic and biotic interactions belowground.

Soil is characterized by its physical, chemical and biological properties and regulates, via feedback mechanisms, the composition, richness and productivity of plant communities. The availability of nutrients is therefore one major factor influencing soil fertility and with that the quantity and composition of belowground soil biota (Wardle *et al.*, 2004), the latter in turn acting again on nutrient availability. Soil microorganisms, in particular soil fungi, have long been thought to secretly hold the reins of plant community processes through their direct and indirect impact on plant–plant and plant–environment interactions by controlling resource cycling along an antagonistic–synergistic interaction continuity. Besides, soil fungi and plants form tight ecological networks with each other; plants have been shown to modulate soil fungal communities (Wardle *et al.*, 2004; Kardol & Wardle, 2010), while soil fungi in turn affect plant growth and community dynamics (Semchenko *et al.*, 2018). Soil fertility is likely to be the stage on which soil fungi act on both plant species richness and productivity, shaping the observable *status quo*. Depending on local habitat conditions and plant–soil–microorganism feedback mechanisms, the composition of fungal communities as actors might change the plant richness–productivity continuum. The role that soil fungi and especially their functional guilds play in the plant richness–productivity relationship is not, however, well known.

High‐throughput sequencing of soil fungal communities and creation of curated databases such as UNITE (Abarenkov *et al.*, 2010) and FUNGuild (Nguyen *et al.*, 2016) have enabled scientists to obtain and assign fungal sequences into expert‐curated species hypotheses (SHs) and further to their potential functions. Using these available tools to divide fungal species into functional guilds (such as saprotrophs, mutualists and pathogens) and acknowledging that these guilds have different associations with plants, Chen *et al*. show that in nutrient‐rich soils there are relatively more of both mutualistic (mycorrhizal fungi) and saprotrophic fungi when plant richness and biomass are higher, whereas in soils with lower nutrient levels mycorrhizal fungi and plant pathogenic fungi are related to plant community richness and biomass (Fig. 1).

**Fig. 1:**
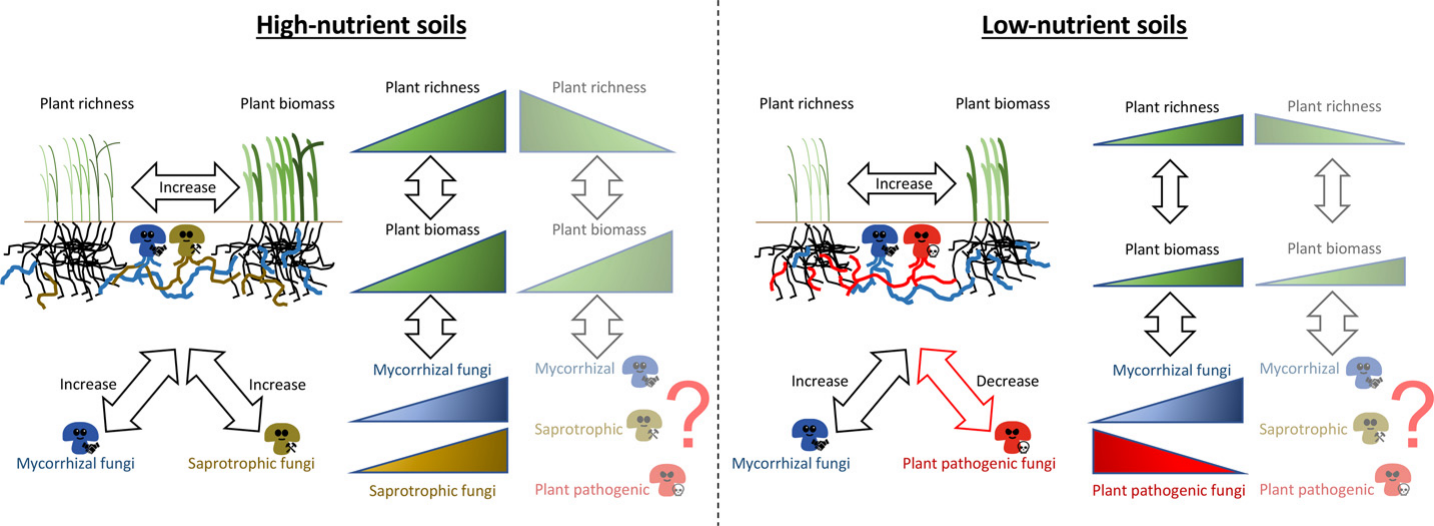
Conceptual model of fungal groups and plant richness–productivity relationship based on the article published in this issue of *New Phytologist* by Chen *et al.* (2020; pp. 1129–1143). In both high‐ and low‐nutrient soils, plant richness and productivity (i.e. plant aboveground biomass) jointly increase and the respective correlation with different soil fungal groups was found by Chen *et al*. However, it remains an open question whether and how the composition of fungal groups changes when plant richness decreases with increasing productivity, and vice versa, in different soil environments. The thickness of arrows indicates the strength of the interaction. The icons depicting fungal groups represent their relationship with plants: mycorrhizal fungi (blue) are cooperating with plants, saprotrophic fungi (olive) promote mineralization processes altering nutrient supplies, and potential plant pathogenic fungi (red) might kill plants.

Van der Putten *et al. *(2016) proposed that plant–soil feedbacks (PSFs), and hence plant communities, would be driven by interactions between soil organisms divided into three categories: symbionts, decomposers and enemies. All types of disturbances are expected to change the balance between fungal functional guilds, and this, in turn, would affect the plant community. Some examples of disturbance driven context‐dependency of interactions between fungal functional guilds are already present (e.g. Grau *et al.*, 2017 in relation to environmental severity in the high Arctic). Chen *et al*. add to this evidence that the balance between fungal guilds is also dependent on the soil nutrient level. In low fertility sites, abundances of the mutualists (mycorrhizal fungi) and saprotrophs were negatively related to each other, suggesting an antagonistic relationship with each other possibly because they need to compete for nutrients. However, the observed positive relationship in high nutrient sites between abundances of the same fungal guilds would potentially indicate a release of competition. Furthermore, only in low nutrient sites there was an interaction between abundances of plant‐pathogens and mutualistic fungi observed, indicating potentially antagonistic interactions between them.

These interactions among soil fungal guilds have further effects on soil processes such as nutrient cycling and decomposition. On the one hand, an increase in richness and (relative) abundance of saprotrophic fungi is likely to promote plant richness and productivity through increases in mineralization and resource partitioning (Bardgett & van der Putten, 2014). On the other hand, plant diversity affects the decomposer community by providing more diverse root‐exudates and litter (Kardol & Wardle, 2010). Similarly, mycorrhizal fungi have been shown to promote plant diversity through facilitation in terms of nutrient uptake and distribution of nutrients among plant species (van der Heijden *et al.*, 2008) but are also dependent in turn on the plant community. The role of plant pathogens in regulating plant diversity is also known in grassland ecosystems, and Mommer *et al. *(2018) demonstrated that fungal root pathogens are an important driver of biodiversity–ecosystem functioning relationships.

Assigning fungi into functional guilds instead of only considering their rough phylogenetic placement (i.e. at the level of phylum or class) is definitely the way forward in understanding how soil‐inhabiting communities affect plant community dynamics and functions. However, typically, roughly half of the sequenced fungal operational taxonomic units (OTUs) can be assigned reliably to species level and for even fewer, function can be assigned. In order to overcome this limitation, efforts to improve databases using cultivation and morphological observation‐based methods, and by adding sequences of known functional groups, are needed.

With the results of Chen *et al*., the intensely debated plant richness–productivity relationship gained another complex yet fascinating aspect of its potential driving mechanisms. Nevertheless, open questions remain, such as the composition of fungal guilds and their influence in states of high plant richness and low biomass, and low richness and high biomass in both high and low fertility soils – habitat conditions that were not studied by Chen *et al.* (Fig. 1). Consequently, it would be highly valuable to extend this study to other herbaceous as well as woody‐dominated natural plant communities around the globe and evaluate its generality. From a conservation point of view, the potential of the discovered role of fungal guilds lies within preserving or re‐establishing plant richness and diversity especially in habitats that have experienced a decrease in either variable. From an agricultural point of view, the study might open doors to a more efficient and increased management of productivity, such as in forest habitats. Certainly, knowledge about the tight connection between soil fungal guilds and plant richness and productivity might help in coping with climate induced abiotic and biotic changes that are yet to come.
